# Neurological Effects of Combining Low Toxic Dose of Tramadol and Nicotine: An Animal Model Evidence of Endoplasmic Reticulum Stress

**DOI:** 10.1155/2023/1953356

**Published:** 2023-08-09

**Authors:** Doaa Ghorab, Ejlal M. Abu-El-Rub, Mohamed Hamdi Gharaibeh, Alaa Yehya, Ramada R. Khasawneh, Laila M. Matalqah, Ahmed Mohamed Helaly

**Affiliations:** ^1^Basic Medical Sciences, Faculty of Medicine, Yarmouk University, Yarmouk, Irbid, Jordan; ^2^Pathology Department, Faculty of Medicine, Mansoura University, Mansoura, Egypt; ^3^Department of Basic Veterinary Medical Sciences, Faculty of Veterinary, Jordan University of Science and Technology, Irbid, Jordan; ^4^Clinical Pharmacy and Pharmacy Practice, Faculty of Pharmacy, Yarmouk University, Irbid, Jordan; ^5^Forensic Medicine and Toxicology Department, Faculty of Medicine, Mansoura University, Mansoura, Egypt; ^6^Clinical Sciences Department, Faculty of Medicine, Yarmouk University, Irbid, Jordan

## Abstract

Tramadol abuse is a common problem in the Middle East in conjunction with smoking. The current study applied immunohistochemistry, western blot, real-time PCR, and ELISA to test the combination toxicity. Low toxic doses of tramadol induced animal brain cortex inflammation and hippocampus injury. Adding nicotine reverted hippocampus pathological changes without triggering marked brain injury. The expression of CHOP protein with real-time PCR showed mild endoplasmic reticulum stress (ER) in rat's brain. Histological, immunohistochemical, and western blotting analysis of CHOP (CCAAT-enhancer-binding protein homologous protein) and BIP (immunoglobulin heavy chain-binding protein) chaperones demonstrated endoplasmic reticulum stress in the brains of animals. Furthermore, the levels of apoptosis and autophagy markers demonstrated a mild reaction. The blood level of serotonin was high in all study groups, with a marked increase in the combined one. The high serotonin levels in the blood can be critical and associated with a high risk of serious withdrawal and pathological consequences. Serotonin receptor blockers such as olanzapine may increase systemic serotonin levels and need further investigation to utterly pinpoint their roles in managing mood disorders. In conclusion, the combination of tramadol and nicotine is less harmful than expected. However, serious withdrawal effects can occur as a result of high systemic serotonin effects.

## 1. Introduction

Tramadol is a centrally acting analgesic drug that is prescribed to treat moderate to severe acute or chronic pain [1]. Nicotine, a lipid-soluble alkaloid, is one of the most accessible and abused chemicals worldwide [2, 3]. Coadministration of cigarettes and opiates has a cumulative impact on many body systems [4]. High rates of morbidity and mortality among regular drug users were recorded [5, 6]. Smokers are more likely to experience chronic pain of greater intensity, a greater number of painful sites, and more associated impairments [7]. Negative impacts on occupational and social functioning have been demonstrated [8]. Compared to nonsmokers, they have higher pain scores and greater demand for opioids during and after surgery [9]. Opiate usage has been reported to encourage smoking habits [10]. A previous study, which included a sample of 48 smokers who are also tramadol addict patients, suggested that tramadol increased the severity of nicotine dependence, as the mean Fagerstrom test for nicotine dependence (FTND) score dropped from 6.67 during the tramadol addiction phase to 4.31 five weeks after stopping using tramadol [11]. In addition, studies have indicated that smokers are more likely than nonsmokers to be on opioid pain medications dependent for longer periods and at greater dosages [12, 13]. Higher levels of reported pain among smokers may also be associated with higher levels of depression, anxiety, and sadness [14].

To explore the molecular underpinnings of nicotine and opioid addiction, several experimental studies were conducted. Azmy et al. [15] reported that nicotine augmented oxidative stress, inflammatory reaction, and possibly apoptosis postcombined exposure to tramadol/nicotine in brain samples of mice. Tramadol abusers were reported to suffer from disrupted fertility profiles [15]. In parallel, previous studies reported that nicotine has a dose-dependent detrimental impact on sperm characteristics [16]. Recently, it was found to induce endoplasmic reticulum stress in the placenta in the rat model [17]. On the other hand, recent experimental work demonstrated the potential protective mechanism of nicotine on injured nerve cells [18]. Despite the induction of oxidative stress, nicotine exerts anti-inflammatory action mediated by alpha 7 nicotine receptors [19, 20]. It was reported that nicotine decreased tumor necrosis alpha and interleukin 1 beta [21].

The endoplasmic reticulum is an essential cell component dealing with protein production, processing, modifications, and transport. Endoplasmic reticulum stress is regarded as a physiological process in addition to pathological marker. If the cell is subjected to an endoplasmic reticulum load, it stimulates the chaperone battery to protect the cell against injury. If the condition is overwhelming, the cell stimulates an apoptosis reaction. BIP(GRP78), an hsp70-related, endoplasmic reticulum-resident protein, and CHOP (CCAAT-enhancer-binding protein homologous protein) proteins are chaperones expressed to protect the endoplasmic reticulum from strain [22].

As a result, nicotine experimentally might have contradictory effects on the brain. Considering these outcomes, this work is aimed at investigating the possible modulatory effects of nicotine on tramadol-induced neuropathological changes in the brain. In addition, it evaluates the expression of specific markers of endoplasmic reticulum stress on exposure to nicotine and tramadol.

## 2. Material and Methods

### 2.1. Animal Groups

Six animal groups were selected for the current study. The animals were 6-week-old male Sprague Dowley rats with 150 to 200 g body weight. The course of the study was a 3-week experiment, and the animals were injected 5 times per week with no injections on the weekends. The experiment was done in the animal house of the Jordan University of Science and Technology Lab. The handling of the animals has been done according to the ethical committee review that accepted the project by code number (752/12/4/86). Each group contained 8 animals. The temperature range was from 21 to 24 degrees Celsius. The animals were kept in hand-made cages feeding on pelleted feed. The animals were evaluated for changes in body weight and sleeping patterns if changed. The animals were decapitated rapidly by the experts in the animal lab to avoid chemical interruption with ER stress data (Table 1).

## 3. Methods

### 3.1. Hematoxylin and Eosin

Firstly, the animals' brains were extracted and fixed with 10% neutral buffered formalin (NBF) and processed by a tissue processor to form paraffin blocks. Three sagittal sections (5 micron thick) were obtained every 200 micron using microtome. Paraffin sections were dewaxed in xylol and hydrated through descending grades of alcohol to distilled water. Then, sections were put in Harris hematoxylin for 5 minutes and washed in running tap water for 5 minutes. The next stage was a differentiation of the tissue sections in 1% acid alcohol (1% HCl in 70% alcohol) for 5-10 seconds. Sections were washed in running tap water again until they became blue. Then, staining in 1% eosin for 10 minutes was carried out and was followed by washing with distilled water. Finally, the sections were dehydrated through ascending grades of alcohol, were cleared in xylol, and were mounted in Canada balsam [23].

### 3.2. Immunohistochemistry

Slides underwent deparaffinization by incubation in the oven at 62-65 degrees Celsius for 30 min; then, they were incubated with xylene and dehydrated in descending grades of alcohol (100%-90%-70%) each for 5 min. The slides of the brain were washed in a buffer solution for 10 min. The next step was epitope retrieval by boiling in a pressure cooker with 0.01 M HIER citrate buffer (heat-induced epitope retrieval) with a pressure cooker for 10 minutes after boiling (pH 6.5) [24]. Blocking of endogenous peroxidase using the 3% hydrogen peroxide in methanol for 5 min was applied. The samples were then washed in the buffer for 5 min; then, the slides with brain sections were incubated with proteinase K 0.04% for 5 min. After that, the slides were washed with PBS for 5 minutes and incubated with rabbit CHOP polyclonal antibody (MyBioSource catalog no. MBS126028) with a dilution of 1 : 500 at room temperature in a humid chamber for 2 hours, and a mouse caspase-8 monoclonal antibody (MyBioSource catalog no. MBS8808615) with a dilution of 1 : 200 overnight and rabbit polyclonal P53BP1 (GenoChem catalog no. GW2740R) with a dilution of 1 : 200 overnight were used. The slides were washed in PBS 3 times for 2 min each, and then 2 drops of the secondary antibody were added and incubated for 10 min. The next step was washing PBS 3 times for 2 min each. Two drops of streptavidin-biotin were added for 30 min at room temperature and washed in PBS 3 times for 2 min each. Diaminobenzidine (DAB) with a dilution of 1 : 50 was added and used as the chromogen for 1-3 min (Abcam rabbit specific HRP/DAB detection kit ab64261). Again, the slides were rewashed in PBS 3 times for 2 min each, and 2 drops of Mayer hematoxylin counterstain were added for 1-3 min. The slides were put in the buffer for 30 seconds, then were washed in distilled water, dehydrated using ascending grades of alcohol, and cleared in xylene for 5 min before mounting [25].

### 3.3. Western Blot

The protein levels of BIP (Cat# MBS857422, Mybiosource, USA), CHOP (Cat# MBS126028, Mybiosource, USA), caspase 8 (Cat# MBS8808615, Mybiosource, USA), and LCIII (Cat# MBS2520564, Mybiosource, USA) were measured by western blotting using species-specific antibodies. Briefly, total protein levels were measured by NanoDrop™ Lite Spectrophotometer (ThermoFisher Scientific) and 50 *μ*g of protein was loaded onto SDS-PAGE. After electrophoresis, proteins were transferred to the PVDF (polyvinylidene difluoride) membrane and incubated with primary antibodies and corresponding secondary antibodies. The membranes were visualized using a gel documentation imaging system (Vilber, France), and bands were quantified using ImageJ software for densitometry.

### 3.4. Real-Time PCR

#### 3.4.1. RNA Isolation

Total RNA was extracted using (Jena Bioscience, Germany) extraction kit. The steps of eluting the RNA were applied to the manual.

### 3.5. cDNA Synthesis

cDNA synthesis kit, which was used in the study, was EasyScript First Strand cDNA Synthesis Supermix Cat. No. AE301.

### 3.6. Real-Time Polymerase Chain Reaction (PCR)

To determine the expression of the target genes, relative quantitative real-time PCR was performed using SYBR-green HOT FIREPOOL EVAGREEN q PCR Supermix from SOLIS BIODYNE. The qPCR cycle protocol is used according to the following.

The initial activation was done once, and the remaining component of the cycle was repeated 40 times. The results were evaluated by the BIO-RAD CFX maestro program. The samples were reevaluated in duplicate to confirm the results (Table 2). The primers were chosen according to (Table 3).

### 3.7. 5-ELISA Technique

The serum samples have been frozen at minus 80 degrees Celsius till examination for ELISA. The samples were tested according to the kit's manual. The methods were obtained from the kits CatLog numbers: ELK8954 for rat serotonin and ELK8953 for rat dopamine kit. All kits were obtained from ELK Biotechnology Company.

### 3.8. Statistical Analysis

Statistical analyses of data were performed using the GraphPad Prism version 9.0 for Windows, (San Diego, California). The *P* values were determined using a one-way ANOVA test. The differences were considered significant if *P* values were 0.05. *t* test has been applied.

## 4. Results

Histological examination has been conducted on different animal groups: G1 high tramadol dose (20 mg/kg day), G2 low tramadol dose (10 mg/kg/day), G3 mixed nicotine/high tramadol, G4 nicotine group 125 *μ*/kg/day, G5 olanzapine 3 mg/kg dose (low toxic dose), and G6 saline negative control group.

**Figure 1:**
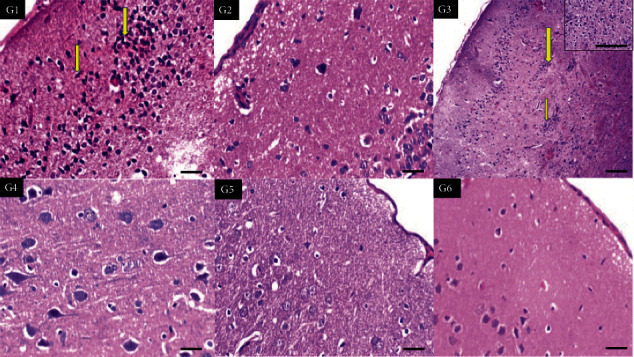
Sections of the brain cortex of six groups showing increased aggregates of inflammatory cells in the cortex of G1 (high-dose tramadol) and G3 (nicotine+tramadol) (yellow arrows) (×400,400,100,400,400,400, respectively).

Mild inflammation was observed in the high-dose tramadol group and the combined tramadol/nicotine-treated group (Figure 1). The combination neither did improve the mild toxic profile nor aggravated it. Lower tramadol, olanzapine, and the negative control were safe on the brain cortex of rats.

**Figure 2:**
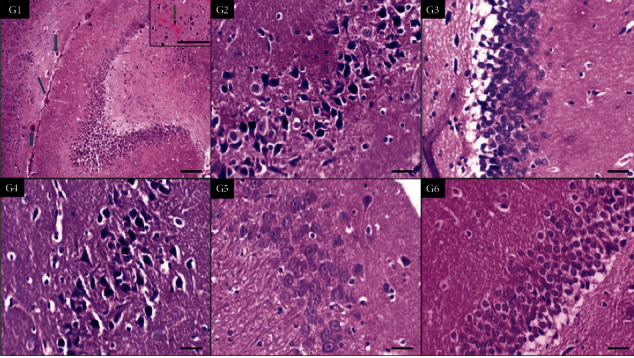
Sections of the brain hippocampus of six groups showing dilated congested blood vessels in G1(high-dose tramadol) (green arrows) and increased degenerated neural cells in G2 (low-dose tramadol) and G4 (Nicotine) (×100,400,400,400,400,400, respectively).

In Figure 2, the histological findings of the hippocampus showed injured neurons in the low tramadol group and nicotine group with vasodilation in the higher tramadol group. This injury is corrected by combining tramadol with nicotine.

**Figure 3:**
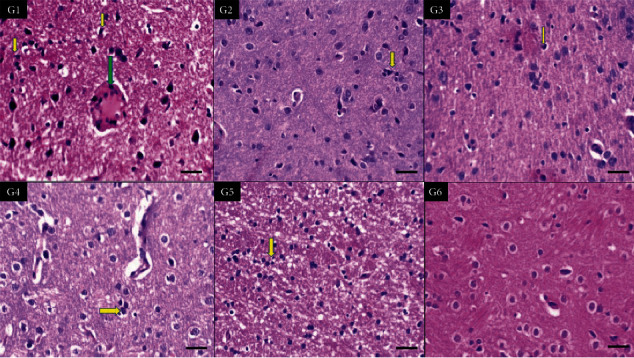
Sections of brain white matter of six groups showing dilated congested vessel in G1(high-dose tramadol) (green arrow) and increased inflammatory microglia in all groups in comparison to G6 (normal control) (yellow arrows) (×400 all).

In Figure 3, the histology showed injured white matter in all nicotine and tramadol groups. Again, high vascularity was demonstrated in higher tramadol dosage.

**Figure 4:**
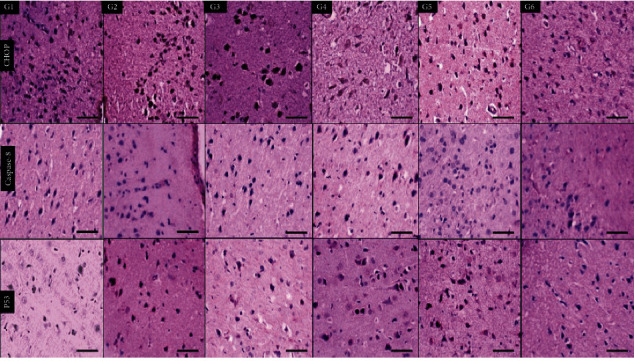
Immunohistochemical staining sections of the brain cortex of six groups showing positive cytoplasmic expression of CHOP antibodies in all groups, including G6 (normal control). Negative expression of caspase-8 antibodies in all groups except mild expression in G4 (nicotine) and G1 (high-dose tramadol). P53 is expressed in all groups with the lowest positivity in G3 (nicotine+tramadol) and G6 (normal control) (×400 all).

In Figure 4, the samples were stained with CHOP immunohistochemical stains where the six groups demonstrated positive staining with different degrees in comparison to G6 (normal control) which shows lower expression. Caspase-8 showed only focal expression in G4 (nicotine) and G6 (normal control), So there was a basal expression of both CHOP and caspase-8 in the brain cortexes of normal control rats.

**Figure 5:**
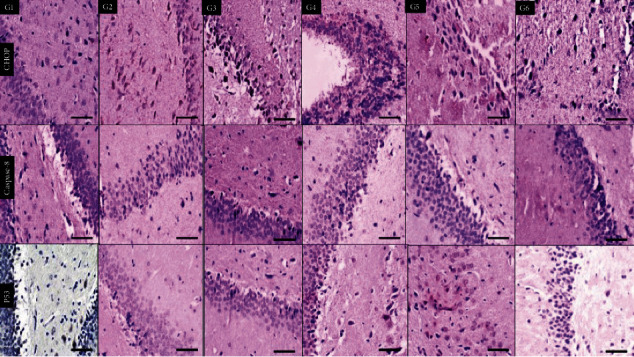
Immunohistochemical staining sections of the brain hippocampus of six groups showing different expressions of CHOP antibodies with the lowest expression in G1 (high-dose tramadol) and G6 (normal control). Caspase-8 antibody is negative in all groups showing only focal positivity in G1 (high-dose tramadol) and G6 (normal control). P53 antibody is negative in all groups except showing mild positivity in G4 (nicotine) and G5 (Olanzapine). (×400 all).

In Figure 5, staining the animal hippocampus showed different expressions of CHOP antibodies. The lowest expression was seen in G1 (high-dose tramadol) and G6 (normal control). Caspase-8 antibody is negative in all groups, showing only focal positivity in G1 (high-dose tramadol) and G6 (normal control). So, there was also a basal expression of both CHOP and caspase-8 in the hippocampus of normal control rats. P53 showed only mild focal positivity in G4 (nicotine) and G5 (olanzepine) with negative all other groups including G3 (nicotine+tramadol).

**Figure 6:**
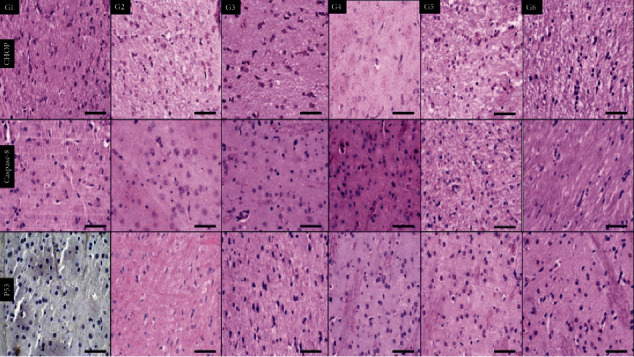
Immunohistochemical staining sections of brain white matter of six groups showing different expressions of CHOP antibody in comparison to negative G6 (normal control). Caspase-8 antibody is negative in all groups except mild expression in G4 (nicotine). P53 antibody is negative in all groups (×400 all).

In Figure 6, all groups showed increased CHOP expression in the white matter in comparison to G6 (normal control). Caspase-8 antibody was negative in all groups except mild expression in G4 (nicotine). P53 antibody was negative in all groups.

**Figure 7:**
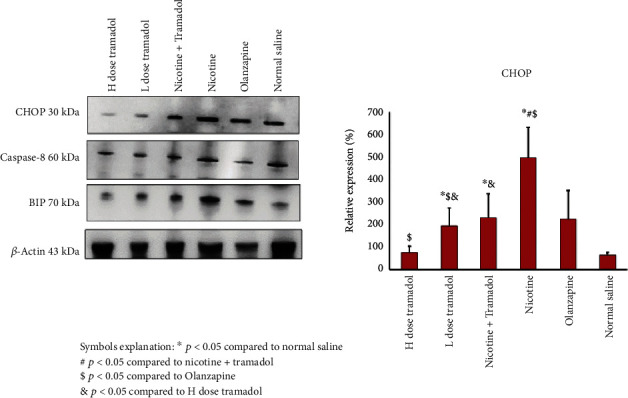
Western blot protein expression of the CHOP endoplasmic reticulum stress (ER stress) marker in rats' brains. The figure also demonstrated Caspase-8 expression, the other BIP ER stress marker, and B-actin as reference protein. As regards CHOP expression, the highest expression was recorded in the nicotine group. The lowest expression was noticed in the high Tramadol group. The combined category showed less CHOP expression than nicotine alone.

Figure 7 showed the western blot expression of CHOP chaperone as a marker of endoplasmic reticulum stress. The experiment tested 5 groups against the negative control group. The groups are 1- high tramadol (20 mg/kg group), 2-low tramadol (10 mg/kg), combined high tramadol/nicotine group, 4- nicotine group 125 mcg/group, 5- olanzapine 3 mg/kg group, 6- saline negative control group. The highest protein expression was in the nicotine group. The negative control also showed a positive expression like a high tramadol dose. The low tramadol group expressed higher CHOP expression; combined nicotine/tramadol expressed a better profile than nicotine alone.

**Figure 8:**
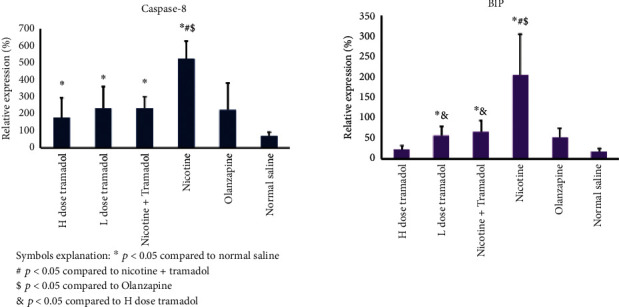
The quantitively expressed caspase 8 and BIP chaperone. The highest expressions were demonstrated in the nicotine group reflecting ER stress in rat brain samples with less expressions in the combined regimen. H correspond to high-dose tramadol and L corresponds to low-dose tramadol.

Figure 8 the BIP chaperone western blot results were supporting findings to the CHOP expression. Indeed, the negative control group has had a basal expression of BIP as well as CHOP. The most abundant expression was the nicotine group. The nicotine and combined groups expressed the two chaperones CHOP and BIP similarly. As regards caspase 8, the highest group was again the nicotine group, and the lowest group was the negative control group. The other groups showed moderate protein expression.

**Figure 9:**
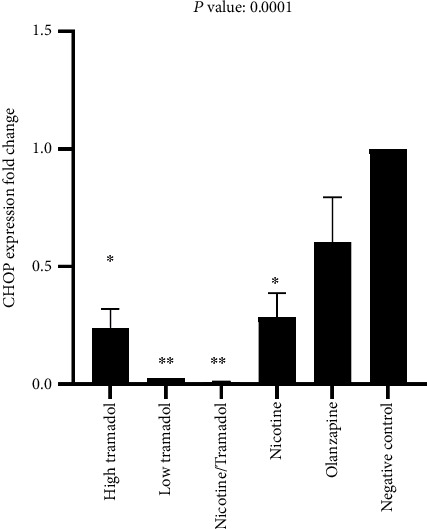
The relative gene expression of CHOP by real-time PCR. The highest expression was the negative control. The combined nicotine/tramadol expression was better than the nicotine group in rat brains.

Figure 9 demonstrated the real-time gene expression of the CHOP gene in the animal rat's brain. The results showed high gene expression in the control and the olanzapine groups. Relatively high expression was recorded in the high tramadol groups, and low expressions were expressed in the low tramadol groups and the combined tramadol/nicotine group. Moderate expression was noticed in the nicotine group.

**Figure 10:**
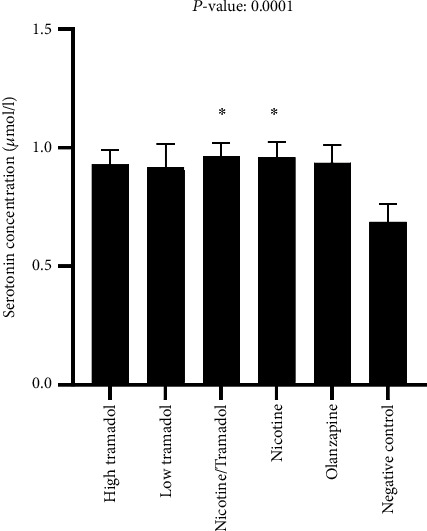
All groups showed statistically significant high concentrations of serotonin levels in the rat blood compared to negative control.

Figure 10 showed the relative serotonin blood level in the different 6 groups. As for serotonin, all groups demonstrated high serotonin blood levels in contrast to the negative control. The highest levels were the nicotine and nicotine/tramadol combination. Also, the other groups, whether tramadol or olanzapine, expressed high blood serotonin levels.

**Figure 11:**
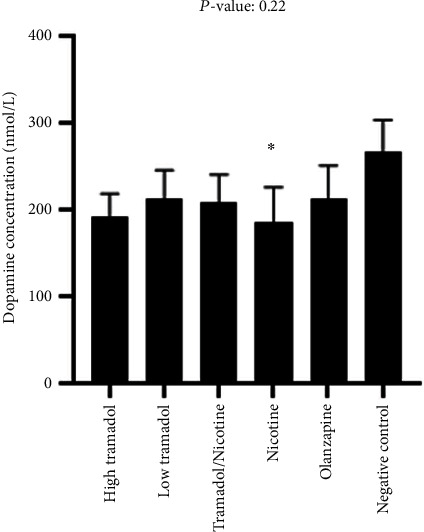
Animals' blood levels of dopamine of the different groups in comparison with negative groups (high tramadol, low tramadol, combined tramadol nicotine, nicotine, and, finally, the negative control). There were no significant changes except in the relation between the nicotine group and negative control evaluated individually by *t*-test in the B component of the figure.

Figure 11 illustrated the relative blood levels of dopamine in comparison to the saline groups. The nicotine group showed only decreased blood levels. These results revealed that nicotine increases serotonin and decreases dopamine in rat blood.

As regards animal fatality, no animal death cases were recorded in the course of the study. The animal group 5 (olanzapine) showed an increase in the body weight of about 20 g per animal, and the rats were sleepy.

## 5. Discussion

Prescription drug abuse, such as tramadol abuse, increases morbidity and death rates, while it also worsens the financial burden of indirect expenditures for health care, health promotion, and surveillance, and dropped economic growth [26, 27]. Prescription and controlled drugs are being used and abused more and more around the world, though the type of drug being abused may vary from country to country [28].

As a centrally acting analgesic, tramadol works through both opioid and nonopioid pathways [29]. Its strong affinity for opioid receptors and suppression of both norepinephrine and serotonin reuptake is what gives it its analgesic effects [30].

Smoking is a major risk factor for opioid addiction [31], and using opioids and tobacco concurrently may improve perceived good effects and satisfaction with drug use, lessen withdrawal symptoms for both substances, and serve as a substitute when one drug is unavailable [32, 33]. Also, smoking enhances opioid usage [34].

Despite the important overlap between nicotine and tramadol, very few experimental researchers have looked into their combined impact. In this study, experimental animal work tracks the low-toxic combination of tramadol/nicotine on rats for 5 days per week for 3 weeks to explore the expected effects on the histology of the brain cortex and hippocampus, immunohistochemical staining, and western blotting for ER stress markers CHOP and BIB chaperones. Western blotting has been applied for caspase 8 reflecting apoptosis and LC3 to detect autophagy [35]. The experiment showed mild ER stress in the brain and demonstrated that both combined nicotine/tramadol did not worsen the brain condition.

Histological examination of the cortex demonstrated an inflammatory response to increasing the tramadol dose. On the other hand, nicotine improved the hippocampus profile. All toxic groups showed injury to the animal's white matter, including toxic olanzapine doses. The vasodilatation was reported to increase with the higher tramadol group. The CHOP immunohistochemistry showed positive staining in all groups. The positive CHOP expression in the control group can be explained by the idea that ER stress is part of the physiological process in the neurons.

The p53 immunohistochemistry profile was the best in the nicotine/tramadol animals.

The hippocampus immunohistochemical staining of CHOP showed improved endoplasmic reticulum function if nicotine was added to tramadol. Minimal response to p53 immune stain was recorded in the hippocampus. Lower cell injury was demonstrated in the combined regimen. It can be said that moderate tramadol use in conjunction with smoking is relatively safe for the hippocampus [36].

Similar work demonstrated tramadol-induced ER stress in the adrenals which was associated with oxidative stress [37]. Furthermore, more studies show that nicotine is a factor inducing ER stress [38]. In this work, the results reveal that both ER-inducing chemicals produce a better ER profile in combination or at least nonadditive.

The positive effects of smoking on tramadol abuse have been seen in other experimental work that supports the current results [39]. The real-time PCR results of CHOP expression were similar but not typical in comparison with western blot results. Both western blot and qPCR showed the combined tramadol/nicotine group demonstrated less ER stress. The discrepancy between the protein expression detection by western blot and qPCR can be explained by the stability of the reference protein actin in comparison with GAPDH.

The BIP protein and caspase 8 expressions detected by western blot were a clear mirror of CHOP expression. Supporting the idea that moderate smoking in conjunction with moderate tramadol abuse appears stable. It is strongly recommended not to deal with both smoking and tramadol abuse in a separate management strategy.

Nicotine and tramadol at such low toxic doses for a relatively short period induced injury to the white matter. Higher tramadol doses showed vasodilation in the animal brains.

Despite the promising histological benefits of mild ER stress, it is dangerous to depend on both smoking and tramadol abuse for longer periods. The ELISA results expressed higher systemic levels of serotonin in animal blood. This hyper serotonin status will end in withdrawal and possible harmful adaptive neural response.

Olanzapine has been used as a positive control of ER stress. The present experiment showed that low-toxic doses of olanzapine promote mild ER stress. Furthermore, the histological profile was safe; however, the dose used in the course was also associated with high blood serotonin despite that the drug is a well-known serotonin blocker. It seems that olanzapine exerts its mood stabilizer effect by a master key common hyper serotonin mechanism.

On the other hand, this phenomenon may explain the withdrawal symptoms of the drug. As regards tramadol, it increases blood serotonin because it is a serotonin reuptake inhibitor. It has been recorded that tramadol abuser suffers from depression postdrug detoxification [40]. Other reports showed that post-tramadol abuse may end in psychosis [41, 42]. In this study, it is recorded that excess serotonin may play a subsequent role in the development of both depression and psychosis. It is hypothesized that olanzapine users do not develop the same fate as tramadol ones because olanzapine blocks the serotonin receptor protecting it from the systemic hyper serotonin status. However, it is postulated to have atypical withdrawal symptoms.

The present data show that the antidepressant effect can be induced by both blocking or stimulating the serotonin receptors, and hyperserotonergic is a cornerstone factor in treating mood disorder rather than serotonin receptor signaling. More research is promising to discover new drugs blocking serotonin receptors and increasing serotonin at the same time. It was hypothesized that serotonin may have a direct effect other than serotonin receptor signaling. It is important to notice that experimental work correlated high blood serotonin and the development of a metabolic syndrome or diabetes with olanzapine toxicity. [43].

On evaluating the blood level of dopamine, the current study elucidates no systemic changes except in the nicotine group. Nicotine is known to increase dopamine release in the nucleus accumbens and substantia nigra. Interestingly, these findings varied in the interspecies responses [44]. It was proposed that tramadol and nicotine would modify systemic dopamine as serotonin. However, nicotine lowered systemic dopamine without effects in other groups. Further work is needed to translate the results to humans.

The limitation of this study is the relatively short period of study. Also, it was needed to add a higher doses group to demonstrate evident apoptotic results. Further studies are needed to track higher doses of tramadol and nicotine for a longer period. More markers of ER stress can be tracked like PREK. Oxidative–inflammation response can be added to future work to study tramadol or nicotine in single or multiple experiments.

The result of this work is a step toward applying the conclusion clinically. As far as I know, little data is available about the level of systemic serotonin in the blood of tramadol abusers or about the ER stress in their brains. Because of this work, we can easily apply the questions to humans. We can track the different ER stress markers on autopsy brains, and we can apply clinical ELISA work to the blood of selected candidates taking tramadol for long periods. All these possible designs cannot be available without the preclinical data from the current work. We have participated in clinical research and recorded that patient abusing tramadol suffered postdetoxification depression. It was not clear why these clinical and comprehensive results occurred. According to the current research, we can answer the question and open a new study for further research. Clinically, according to expert opinion, we noticed that many drivers in Egypt and possibly in the Middle East abuse tramadol in conjunction with smoking for a long period of time and look stable. As a result, we created this model to support the expert data. The combination of clinical and experimental work could explain the mechanisms of human diseases depending on different organism models, from bacteria to large animals.

## 6. Conclusion

The current study was able to explore patients abusing moderate doses of tramadol and smoking for a relatively short period. The present model showed that tramadol, nicotine, or the combination of two substances promotes mild ER stress. The combination is relatively safe with the potential of withdrawal syndrome like mood and psychotic seqala. Further studies are needed to comprehend the role of serotonin and olanzapine in managing mood disorders. The present study was performed in rats, and the results should be correlated or extrapolated to humans.

## Figures and Tables

**Table 1:** The different animal groups of the study and both the route and the dose of chemicals used.

Group	Treatment/dose	Route
1	Tramadol 20 mg/kg	Oral gavage
2	Tramadol 10 mg/kg	Oral gavage
3	Tramadol 20 mg/kg /nicotine 125 *μ*/kg	Tramadol oral gavage/nicotine subcutaneous
4	Nicotine 125 *μ*/kg	Subcutaneous
5	Olanzapine 3 mg/kg	Oral
6	Saline	Oral

**Table 2:** The cycle of real-time PCR for CHOP, BIP, and GAPDH gene expressions.

Initial activation	95 degrees Celsius	12 min
Denaturation	95 degrees Celsius	15 sec
Annealing	62 degrees Celsius	30 sec
Extension	72 degrees Celsius	30 sec

**Table 3:** The primers of CHOP and GABDH for qPCR reaction.

Gene	Forward primer	Reverse primer
CHOP	GAAAGCAGAAACCGGTCCAAT	GGATGAGATATAGGTGCCCCC
GAPDH	CCTTCATTGACCTCAACTAC	GGAAGGCCATGCCAGTGAGC

## Data Availability

All data is available.
